# Editorial: Nanomaterials for biology and medicine

**DOI:** 10.3389/fchem.2023.1244175

**Published:** 2023-08-17

**Authors:** Guannan Wang

**Affiliations:** College of Medical Engineering and the Key Laboratory for Medical Functional Nanomaterials, Jining Medical University, Jining, China

**Keywords:** nanomaterial, nanoprobes, nanoenzyme, biology, medicine

The desire for novel technology applications in biology, chemistry, engineering, physics, and medical science has fueled nanomaterials research. Nanomaterials have risen to prominence in technological breakthroughs due to their adjustable thermal, mechanical, electronic, and biological properties and superior performance over bulk equivalents (Baig et al., 2021; Mazari et al., 2021). Accordingly, numerous strategies have been projected to construct various functional structures by the integration of various types of nanomaterials, ranging from metals, metal oxides, alloys, and semiconductive materials to multifarious inorganic and organic polymers (Hao et al., 2010; Wagner et al., 2019). Specifically, nanomaterials offer the virtues as the therapeutic and diagnostic tools, which is owing to their small sizes, design flexibility, large specific surface area, and simple surface modification to enhance avidity for the targeting molecule (Yu et al., 2021; Qiao et al., 2022; Pourmadadi et al., 2023). There has been an explosive development in the use of nanomaterials for biomedical applications to probe biological processes, diagnose and treat medical conditions. The distinguishing characteristics of nanomaterials enable them to preferentially penetrate and be retained by cells and tissues of living organisms, thereby achieving developments in new approaches to target in biological cells and biological tissues. Additionally, the size scale of nanomaterials will provide the inspiration to build complexity into the nanoprobes that endow them versatility with both diagnostic, therapy, and drug delivery function. Moreover, diversified surface modification strategies have broadened the application of nanomaterials for biomedical applications, which adds the property of stability, biocompatibility, biodistribution, solubility, biological or therapeutic effects (Sztandera et al., 2019; Liu et al., 2021).

In this Research Topic, the authors were invited to contribute their research works, which enable the better explanation on the recent advances in diversified applications of nanomaterial in the biology and medicine. Yu et al. have contributed a literature review on the current progress on the nanomaterials’ application in diagnosing and treating of glioblastoma (GBM). They discussed the nanomaterials-based nano-diagnosis or treatment mechanisms. Additionally, the advanced application progress of nanomaterial combination diagnostic and therapeutic tools for GBM was summarized. Nano-catalytic therapy, acting as an innovative strategy, has been extensively explored. Shi et al. group synthesized a multi-functional magneto-gold nano-enzyme AuNC@Fe_3_O_4_ and evaluated their anti-cancer ability in the hepatocellular carcinoma (HCC cells) *in vitro*. The preparative nano-enzyme AuNC@Fe_3_O_4_ with a small size was characterized using various techniques and demonstrated with high peroxidase (POD)-like activity, good photothermal conversion efficiency, and can inhibit cell proliferation and enhance cell apoptotic ability in cancer cells, providing a potential anti-cancer method for HCC. Responding to the oxidation state of the micro-environment of bacterial infection, Dorma Momo et al. explored a near-infrared (NIR) photothermal bacterial inactivation by reasonably designing a Metal organic Framework (MOF)-based nano-composite, offering a novel inspiration for constructing precise nano-therapeutic systems. They systematically studied the strong deactivation effect on the Gram-negative and -positive bacteria and intense therapeutic effectiveness on the mouse skin wound infection model of the designed nano-system.

Native and synthetic nanomaterials have drawn a lot of research interests and been projected as the key components for construction of drug delivery systems for healing patients in clinical. Ravelo-Nieto et al. constructed a cellular drug delivery system by using silanized fullerenol and silica nanoparticles (SN) as the nano-structured supports to conjugate potent cell-penetrating agents. The nano-bioconjugates showed distinct intracellular trafficking and endosomal escape behavior in the cell lines, which indicated the potentiality to address the challenge of cytoplasmic drug delivery and the development of therapeutic methods for lysosome storage disease. You et al. developed a monodispersed and biocompatible mesoporous SN (MSN) divergent porous channel for loading dapagliflozin (DAPA). They constructed a drug delivery system through the surface-modification of the cardiac-targeting peptides to release drug for the hypoxic and weak acid damaged cardiomyocytes. This MSN-based nanocarriers for the DAPA delivery system can achieve the efficacious cardiac repair and regeneration *in vivo*. Gui et al. research group exploit a pH responsive antibacterial delivery system (Imi@ZIF-8) for the antibiosis treatment of *A. baumannii*. They found that at an imipenem concentration of 10 mg/kg, the Imi@ZIF-8 nano-system manifested the outstanding therapeutic efficacy against *A. baumannii* in the mice with celiac disease.

Intended as a one-stop reference, this Research Topic provides the reader with the most-up-to-date and comprehensive exploration of a variety of the nanomaterial applications for biology and medicine. Briefly, these nanomaterials can be applied to perspective to advance in intrinsic nano-therapy across the biomedical area, from cancer therapeutics to microbial infection treatment and tissue regeneration. As such, this Research Topic provides the most comprehensive coverage of this intriguing field of study. Currently, this Research Topic has published 11 articles and received over 2,900 downloads and 14,000 readings worldwide.
